# Assessing the Saudi Ministry of Health Instagram messaging through the lens of the health belief model

**DOI:** 10.3389/fpubh.2025.1683619

**Published:** 2026-01-09

**Authors:** Sharifah AlDossary, Mashael Alzaid, Hadeel Albedewi

**Affiliations:** 1 College of Public Health and Health Informatics, King Saud bin Abdulaziz University for Health Sciences, Riyadh, Saudi Arabia; 2 King Abdullah International Medical Research Center, Riyadh, Saudi Arabia; 3 Ministry of National Guard - Health Affairs, Riyadh, Saudi Arabia

**Keywords:** communication, COVID-19, extended parallel processing model, health belief model, Instagram, pandemic, public engagement, public health

## Abstract

**Background:**

Public health authorities are recognizing the importance of social media platforms as a way to spread health messages, engage and enhance communication with the public, and improve the promotion of programs, products, and services. However, the overabundance of information and the spread of misinformation, disinformation, and misleading news, images, and videos in social media have led to an “infodemic.” This calls for analyzing the messages communicated by governmental channels to improve public trust and increase public compliance.

**Objectives:**

This study aimed to explore how the Saudi Ministry of Health (MOH) used Instagram to communicate with the public during the COVID-19 vaccination-promotion phase and to understand the key constructs influencing public engagement.

**Method:**

All Instagram posts from the Saudi MOH official Instagram account related to COVID-19 vaccine and corresponding likes and comments between December 12, 2020, and June 13, 2022, were extracted. Content analysis was performed following the health belief model (HBM) and the extended parallel processing model (EPPM) key constructs: perceived susceptibility, perceived severity, perceived benefits, perceived barriers, and cues to actions/efficacy. The median public engagement was measured by examining the number of likes and comments for images and videos. The association between the key constructs and public engagement was assessed using a Mann–Whitney U test.

**Results:**

A total of 258 Instagram posts were included in the analysis. The most frequently used HBM key constructs were cues to action/efficacy (75%) and perceived benefits (60%). The remaining HBM constructs were found in less than 25% of the Instagram messages. Only 21% of the Instagram messages followed the EPPM construct. Overall, the median public engagement was not associated with the HBM and EPPM constructs.

**Conclusion:**

The Saudi MOH can improve its communication strategy by including a proportional number of HBM key constructs, and increasing the number of Instagram messages with EPPM constructs in future public health promotions.

## Introduction

Health promotion activities and education through social media offer direct access to health information, enabling networks of support among individuals (1). Public health authorities are recognizing the importance of social media platforms as a way to spread health messages, engage and enhance communications with the public, and improve the promotion of programs, products, and services (2, 3). There is an increasing amount of research on how social media platforms can be used in public health communication, with the majority focusing on X, formerly known as Twitter (4). Unlike X, which was developed as a text-based platform, Instagram was designed as a visual-based social media platform that uses images and videos rather than text. There is a higher probability of drawing attention and increasing user recall through visuals than through text alone (5, 6). As of 2025, Instagram ranks as the third most popular social networking platform with 2 billion monthly active users worldwide (7). Saudi Instagram users account for 76.40% of the entire population and represent 26.80 million users (8). In addition, Instagram is used by many institutions, such as non-governmental organizations (NGOs) and health entities, as a channel for communication with the public (9).

During the coronavirus disease (COVID-19) pandemic, public health authorities had to follow measures, such as wearing face masks, hygiene, social distancing and vaccination, to control the pandemic and influence individuals’ behaviors within a brief period (10, 11). However, vaccination hesitancy and a lack of confidence by the public were apparent, despite access to vaccine services and formal requests by many governments (12, 13). Many studies have revealed several factors related to public vaccine acceptance, including concerns about the safety of the vaccine, lack of knowledge among the community, and lack of trust in the healthcare system (14). The overabundance of information and the spread of misinformation, disinformation, and misleading news, images, and videos on social media have led to an “infodemic,” a term resurfaced by the World Health Organization (15). This infodemic is leading to vaccine mistrust and hesitancy, creating a need for public behavioral changes. Therefore, analyzing messages communicated through governmental channels is warranted to increase public compliance (16, 17).

Among various conceptual frameworks for health behavior, the health belief model (HBM) is the most widely used to guide, predict and explain health behavioral changes (18, 19). The key constructs of HBM include perceived susceptibility, perceived severity, perceived benefits, perceived barriers, cues to action and self-efficacy. These constructs collectively contribute to predicting whether people will take action to prevent, control, and screen for diseases (20–23). Furthermore, the extended parallel processing model (EPPM) captures aspects of HBM to explain how message components combining perceived threats (perceived severity, perceived susceptibility) and efficacy can result in either adopting a protective behavior or rejecting it (24). A two-way process can result from combining high threats with high and low efficacy: (1) When high threats and high efficacy are present; this will lead to danger control and message acceptance. (2) When high threats and low efficacy are present; this will lead to fear control and message rejection (25). Other behavioral frameworks such as the Theory of Planned Behavior (TPB), Protection Motivation Theory (PMT), and the Risk Information Seeking and Processing (RISP) model mainly address self-reported intentions and information seeking behaviors. These are less suited for analyzing message content on social media. In contrast, the HBM provides observable constructs, while the EPPM complements it by highlighting the interaction between threat and efficacy, making their combined use a coherent approach for evaluating governmental health communication (26–30).

Several studies have used HBM and EPPM to analyze messages on visual-based social media platforms in Zika virus, COVID-19, enteric illness, and breast cancer (4, 28–36). Findings showed an uneven use of HBM constructs across studies (31–34, 36). The results emphasized the importance of including more combinations of constructs, namely, combining severity and efficacy in risk communication, to increase the adoption of a recommended behavior (28, 29). A study by Alkazemi et al. examined 1,000 Instagram posts from member states of the Gulf Cooperation Council’s (GCC) Ministries of Health Instagram accounts through the lens of the HBM (37). The study recommended including a proportional amount of HBM’s key constructs to facilitate more effective communication with the public. Despite this, significant research gaps remain. Instagram’s visual nature and extensive user base make it uniquely suited to deliver health messages that engage emotion, enhance attention, and improve message recall, through a content delivery format distinct from text-based platforms like X (formerly Twitter) (35, 38–41). While multiple studies have analyzed governmental health communication on X, no published research has assessed how the Saudi Ministry of Health utilized Instagram during the COVID-19 vaccination campaign, or how message framing influenced public engagement (42–45). Addressing these gaps is essential to improve evidence-based communication strategies and optimize public engagement during future health emergencies.

By addressing this gap, the study not only contributes to enhancing digital risk communication on visual platforms, but also aligns with the broader global health objectives. Following the WHO competency framework for managing infodemics, this study can help us better understand and manage infodemic interventions in Saudi Arabia and provide us with a way to strengthen the resilience of communities (46, 47). The aim of this study is to analyze Instagram messages by the Saudi Ministry of Health (MOH) during the COVID-19 vaccination-promotion period through the lens of HBM and EPPM constructs and how these messages influenced public engagement.

## Methods

### Study setting

This content analysis aimed to explore how the MOH used its Instagram account during the COVID-19 vaccination-promotion campaigns in Saudi Arabia. The MOH’s official Instagram account (@Saudimoh) reports health-related information and is updated on a regular basis. The study sample included COVID-19 vaccine-related Instagram messages published by the Saudi MOH’s verified account, from December 12, 2020 (first post related to the COVID-19 vaccine) to June 13, 2022 (last post released related to the COVID-19 vaccine). To be included in the analysis, Instagram messages had to be in the form of an Instagram post (not a highlight or a story) and written in Arabic. Instagram posts that are not related to COVID-19 vaccination-promotion campaigns were excluded. This includes posts about other coronavirus precautionary measures, such as social distancing, hand hygiene measures, and wearing the recommended facial masks. Furthermore, Instagram messages related to other health promotion activities were excluded. All posts meeting the criteria within the defined timeframe were included.

### Data collection

Data was collected from the official Saudi MOH Instagram account (@saudimoh). Instagram posts are publicly displayed for the population; therefore, no official request or approval was needed to extract the data. The data extracted and analyzed from the Instagram messages include: the target audience (older adults, public, pregnant women, adolescents and children), the vaccine dose sequence (general, first, second, booster, and second booster doses), the use of hashtags in the post or caption, and the type of media (images, videos/reels), the number of likes and comments per post. Consequently, the engagement rate was calculated using the following formula: The number of public interactions (number of likes + number of comments with a post)/divided by the number of account followers and multiplied by 100 (48).

### Content analysis and coding categories

This study used both qualitative and quantitative analysis methods. The selected Instagram messages were coded according to the HBM’s key constructs and applied in the messages as: (1) perceived susceptibility, the message content defined the population at risk and their risk levels, personalized risk based on a person’s trait or behavior, and heightened perceived susceptibility if low, (2) perceived severity, the message content described and specified consequences of the risk and condition, (3) perceived benefits, the message content clarified the positive effects to be expected, described the evidence of effectiveness, and defined the actions to take: how where and when, (4) perceived barriers, the message content addressed and reduced barriers through reassurance, correction of misinformation, and assistance. (5) cues to action/self-efficacy, the message content promoted awareness, and provided reminders, how-to information, motivation, training, and guidance in performing actions. The constructs were coded following a codebook presented in Supplementary material 1. Coding was applied to the visual content of posts, including any text appearing within the image or video frame. The constructs “cues to action” and “self-efficacy” were combined into a single category, this decision was informed by the overlapping function of these constructs in motivating behavior and has been adopted in previous content analysis research on social media-based health promotion (29, 49). A construct was coded as ‘present’ if it appeared in the visual content. Coding was not mutually exclusive, meaning that a single post could be assigned multiple constructs when applicable. The coding protocol was developed and tested during the coding process. After the coding protocol was finalized, the reliability of the coding was measured using Krippendorff’s alpha (*α*). Intercoder reliability was established by two independent coders (MA and SA). Each author independently coded a randomly selected subsample of 40 (15%) of the Instagram posts. This percentage meets Neuendorf’s recommendation to code 10–20% of the total selected sample for reliability agreement (50). Reliability was evaluated using the ReCal 0.1 statistical program and Cohen’s Kappa (51). The k values of the two variables “perceived benefits” and “perceived severity” were greater than 0.8 which indicate almost “perfect agreement.” The k values of the two other variables “cues to actions/self-efficacy” and “perceived susceptibility” were 0.78 and 0.65, respectively, which indicate “substantial agreement,” and the value of one variable “perceived barrier” was 0.59 which indicate “moderate agreement” (52). The coding inconsistencies in the “perceived barrier” variable were resolved through discussion. After intercoder reliability was established, the first coder (MA) coded the remaining Instagram posts.

### Statistical analysis

Descriptive analysis was used to measure the frequency and percentages of the categorical data. Non-parametric Mann–Whitney U test was used to check for differences in Instagram engagements (likes and comments) between posts with or without the HBM and EPPM key constructs and vaccine doses, and to check for differences of the public engagement rates in different media types. The statistical analysis was performed using IBM Statistical Package for Social Sciences (SPSS) statistics software (Version 29).

## Results

The Saudi MOH official Instagram account posted a total of 331 messages related to coronavirus disease during the COVID-19 vaccination-promotion campaign. Overall, 258 (77.9%) of the Instagram messages were included in the analysis. The other 73 (28%) messages were excluded for not meeting the inclusion criteria. The general characteristics are presented in Table 1. The most frequent media types used in Instagram messages were images (*n* = 200, 77.5%). The total number of Instagram messages with hashtags in the captions or posts was 110 (42%). The most frequent Instagram messages were targeted toward the general public with a frequency of 207 (80%). The most frequent Instagram messages mentioned COVID-19 vaccine in general without specifying the dose order (*n* = 164, 63%).

**Table 1:** Summary characteristics of Instagram messages.

Characteristics	n (%)
Media type	
Images	200 (77.5%)
Videos	58 (22.5%)
Hashtags	
Yes	110 (42.6%)
No	148 (57.4%)
Target groups	
General public	207 (80%)
Elderly	20 (7%)
Children	17 (6%)
Adolescent	8 (3%)
Pregnant women	6 (2%)
Covid-19 vaccine dose sequence	
General	164 (63%)
First dose	32 (12%)
Second dose	19 (7%)
Booster: First dose, Second dose	28 (10%), 2 (0.8%)
Others: No vaccine	13 (5%)
HBM key constructs	
Cues to action/efficacy	195 (75%)
Perceived benefits	156 (60%)
Perceived barriers	63 (24%)
Perceived susceptibility	50 (19%)
Perceived severity	37 (14%)
HBM key construct per post	
1 key construct	87 (33%)
2 key constructs	119 (46%)
3 key constructs	34 (13%)
4 key constructs	16 (6.2%)
5 key constructs	2 (0.8%)
Threats and efficacy/cues to action	
Severity and cues to action/efficacy	18 (6.9%)
Susceptibility and cues to action/efficacy	28 (10%)
Severity, susceptibility, and cues to action/efficacy	11 (4.2%)
Total	57 (21.1%)

Across all the HBM key constructs, the cues to action/efficacy was the most used (*n* = 195, 75%), followed by the perceived benefits of the COVID-19 vaccine (*n* = 156, 60%), as listed in Table 1. When examining the most common number of combinations in an Instagram post, the most common combination found was two HBM key constructs per post (*n* = 119, 46%), followed by one HBM key construct per post (*n* = 87, 33%). Efficacy/cues to actions and threats (perceived severity, perceived susceptibility) were examined in combined Instagram messages (images, videos/reels, and captions) to assess the EPPM constructs in which the risk messages should initiate the danger control process. The most frequent combination was the susceptibility and cues to action/efficacy (*n* = 28, 10%). The second most frequent combination was severity and cues to action/efficacy (*n* = 18, 6.9%), and the least frequent combination was severity and susceptibility and cues to action/efficacy (*n* = 11, 4.2%). The total Instagram messages on threats and efficacy/cues to actions were (*n* = 57, 21.1%), as listed in Table 1.

According to the engagement rate formula, the median engagement rates for images and videos were 0.198% and 0.100%, respectively. A Mann–Whitney U test was used to compare the median public engagement rate according to the media type (images vs. videos). The median public engagement rate was significantly higher in images as compared to videos (*p* = 0.002). Additionally, the median numbers of likes and comments were significantly higher when the hashtags were absent (U = 6,066 and 5,725, respectively; *p* < 0.001 for both).

Analysis using a Mann–Whitney U test revealed that the median number of likes was significantly higher when the perceived benefits key constructs were missing than when they were present (*p* = 0.031). There was no significant difference in the number of likes and comments in the remaining HBM key constructs. Additionally, the presence of efficacy/cues to actions and threats in Instagram messages revealed no significant association with the median public engagement (Table 2).

**Table 2:** Public engagement for Instagram messages with or without HBM and EPPM construct.

HBM key construct	Public engagement	Presents	Absent	U	*p*
Perceived benefits	Like	1,547	1992	6,695	0.031*
	Comments	153.2	152	2,563	0.101
Cues to action/efficacy	Like	1,697	1,571	5,805	0.513
	Comments	153.5	152	5,595	0.134
Perceived barrier	Like	1,595	1,693	6,090	0.919
	Comments	183	143	5,505	0.29
Perceived susceptibility	Like	1,451	1707	3,451	0.129
	Comments	155	197	5,072	0.828
Perceived severity	Like	1,407	1728	3,360	0.083
	Comments	155	152	4,057	0.976
Threats and efficacy/cues to action	Like	1,457	1722	4,237	0.164
	Comments	166	149	4,352	0.711

Numbers are presented as median. * denotes a p value less than 0.05.

Analysis using Mann–Whitney U test revealed that the median number of likes was significantly higher for posts mentioning the first dose of COVID-19 vaccine (*p* = 0.028). In contrast, the median number of likes was significantly lower in the posts including the booster dose of COVID-19 vaccine (*p* = 0.023). There was no significant difference in the median comments of any of the dose sequence variables (Table 3).

**Table 3:** Public engagement for Instagram messages with or without dose sequence.

Vaccine dose	Public engagement	Presents	Absent	U	*P* value
The general vaccine	Like	1703	1,505	6,169	0.365
	Comments	155	158	6,333	0.606
The first dose	Like	2,467	1,597	2,746	0.028*
	Comments	178	152	2,858	0.058
The second dose	Like	914	1,693	1803	0.135
	Comments	77	153	1976	0.361
The booster dose	Like	1,430	1703	2,545	0.023*
	Comments	166	152	3,357	0.901
No vaccine	Like	1,268	1,693	1,411	0.489
	Comments	111	155	1,159	0.102

Numbers are presented as median. * denotes a p value less than 0.05.

## Discussion

In this research, we examined the HBM key constructs and EPPM construct within the Saudi MOH’s Instagram posts along with the likes, comments, and public engagement rates during the COVID-19 vaccination-promotion phase. Our research found that the HBM key constructs were not used in equal proportions, as the most used HBM key constructs were the cues to action/efficacy (75%) and perceived benefits (60%), with more than half of the Instagram messages containing them. In contrast, the remaining HBM constructs, including perceived barrier, perceived susceptibility, and perceived severity were found in less than 25% of the Instagram messages during the study period. These messages focus more on the management of health threats (cues to actions and perceived benefits) and less on the perceived threats (perceived severity and perceived susceptibility) of the COVID-19 virus. This is a concern because for the public to follow preventative measures, they need to not only understand the benefit of the COVID-19 vaccine and get guidance/motivation concerning the COVID-19 vaccine services but also consider the severity and susceptibility of the coronavirus disease. The higher representation of cues to action/efficacy and perceived benefits compared to the remaining constructs aligns with findings from another study done in 2018 to analyze the GCC nations’ ministries of health Instagram accounts (37). The study demonstrated that cues to actions and perceived benefits of HBM key constructs were mentioned in more than 20% of the Instagram posts, while all other constructs were only present in 6% of the posts (37).

Furthermore, we examined the number of HBM key constructs in combination. Our findings reveal that the most common combinations are two HBM key constructs per post (46%). The total percentage of Instagram posts with combinations of HBM key constructs was 66%. This study shows an overall good level of HBM construct combinations per Instagram post. On the other hand, only 21% of the Instagram messages followed the EPPM construct (threats and/cues to action and efficacy) during the COVID-19 vaccination-promotion phase. These results are consistent with a Canadian Instagram content analysis study during the COVID-19 pandemic which found that Instagram messages rarely included these combinations across different news media, influencer categories, and science communicators (29). This raises a concern, as the EPPM suggests that the combination of threats and cues to action/efficacy constructs will initiate the danger control process, which will affect the behavior, attitude, and intentions of the message receiver (53). When the threats are not properly expressed in a message, even if the efficacy information is present, a low act of motivation results. However, messages containing low efficacy and high threat information lead to fear and message rejection. Even more, when both threat and efficacy information are absent in a message, the threat is not considered, and the message is irrelevant (53). Another Canadian study related to COVID-19 found that messages targeting both efficacy and threats were highly associated with low fear, and public adherence to governmental recommendations (54). These messages need to be carefully crafted and designed to be accepted by the receiver and to increase compliance with a recommended behavior (29). This is especially true for public health entities designing interventions and communication strategies to improve user engagement on social media platforms.

The analysis of public engagement in relation to the HBM and EPPM constructs showed significantly higher median number of likes when the perceived benefit construct was absent. This was the only HBM construct found with a significant difference in the median public engagement. Overall, the median public engagement was not associated with the HBM and EPPM constructs. These results can be explained by the uneven proportional amount of the HBM construct, and the low amount of EPPM construct found per Instagram post during the study period.

The analysis of median public engagement in different dose sequence revealed a significantly higher median number of likes in posts mentioning the first dose of COVID-19 vaccine (*p* = 0.028). Furthermore, the first dose of the COVID-19 vaccine was the most frequently mentioned (*n* = 32, 12%) compared to the other dose sequence variables. This reflects the Saudi MOH’s major efforts at the beginning of the COVID-19 vaccine-promotion campaign to push Saudi citizens and residents to get their first dose of the COVID-19 vaccine. Moreover, the median number of likes was significantly lower in the booster COVID-19 vaccine related posts (*p* = 0.042). This indicates a decrease in public engagement as the COVID-19 vaccine dose sequence messages increased in dose order. It emphasizes the role of timing in public health communication as public engagement peaks during certain phases of health campaigns (55).

The median public engagement rate was significantly higher (*p* < 0.001) when the Instagram messages did not include hashtags. These findings correspond with a previous content analysis done on Instagram where hashtags were negatively associated with public engagement (56). A possible explanation is that hashtags could be perceived as unauthentic by the public (57). However, other content analyses of X platform posts found a positive association between hashtags use and public engagement (42, 58). This suggests tailoring health communication strategies depending on the social media platform used. A recent study on using social media for public health awareness identified specific patterns of engagement unique to each platform (55).

The analysis of public engagement rate in association with images and video/reels showed significantly higher public engagement in images than video/reels (*p* < 0.002). Images were also the most used media type (200, 77%) during the study period. These results correspond with another study, confirming that the most used media type in Instagram was images, and revealing that images had a strong significant positive relationship with engagement, while videos/reels public engagement was insignificant by comparison (59).

Our study has a few limitations. First, the inclusion of only one social media platform, Instagram, as public health entities use their health promotion activities in different social media platforms to spread their message. Second, we were unable to access the followers’ demographics since the MOH’s Instagram account restricted the followers’ access to the public. The followers’ demographics can help us further analyze the audience age group, gender, region, and their association with the Instagram message variables and public engagement. Additionally, this study did not segment messages by specific time phases of the pandemic. Although posts were categorized by vaccine type (general, first, second, and booster doses), the analysis did not explicitly examine time or policy related variations. Future research could explore phase specific analyses to better understand potential differences in communication focus and public engagement. For future recommendations, a similar study can be conducted on other social media platforms with MOH verified accounts, such as X and Facebook. Additionally, a sentimental analysis can be conducted on the comments section to further understand the public’s emotional responses to the messages, as this is an important factor in assessing the effectiveness of the communication strategy. This would allow for a better understanding and management of the infodemic, with the ultimate goal of avoiding misperceptions and promoting clarity, thereby enabling individuals to take the appropriate preventive measures.

## 5 Conclusion

The study provided some insight into the Saudi MOH communication strategy during the COVID-19 vaccination-promotion phase. Our results identified that the Saudi MOH Instagram messages focused on the management of health threats and less on the perceived threat, and as result, a low amount of Instagram messages with EPPM constructs (threats and/cues to action and efficacy) were found. For future health promotion campaigns, it is recommended to include a proportionally even amount of HBM key constructs and increase the amount of Instagram messages with EPPM constructs. These recommendations might improve the level of public engagement and influence the behavior of the general public to take the recommended health-related preventative action.

## Data Availability

The raw data supporting the conclusions of this article will be made available by the authors, without undue reservation.
