# Supplementary material for: Assessing the Saudi Ministry of Health Instagram messaging through the lens of the health belief model
Source: Front Public Health. 2026 Jan 9;13:1683619. doi: 10.3389/fpubh.2025.1683619 (PMC12827744; doi:10.3389/fpubh.2025.1683619)
Supplement: Supplementary file 1 [file Table_1.DOCX]

Supplementary Material

# Supplementary Data

**Code book:** The HBM key construct definitions, Applications, and examples in the Instagram messages.

| HBM key constructs | Definition | Application  (The message content) | Examples  (The message content) | Translated Examples |
| --- | --- | --- | --- | --- |
| *Perceived severity* | refers to the understanding that contracting the disease or leaving it untreated will lead to serious medical, clinical, and social consequences. | The message content describes and specifies consequences of the risk and condition. | "كورونا (كوفيد – ١٩) قد يتسبب بمضاعفات خطيره " | "COVID-19 may cause serious complications." |
| *Perceived benefits* | The individual must believe that taking action to engage in preventative behavior will provide positive outcomes and reduce threats. | The message content Clarify the positive effects to be expected.  Describes evidence of effectiveness  Define action to  take:  how, where, and when. | “ دراسة تؤكد الجرعة التنشيطية توفر حماية بنسبة ٩٠٪ ضد أعراض كوفيد – ١٩ عند كبار السن " | "A study confirms that the booster dose provides 90% protection against COVID-19 symptoms in the elderly." |
| *Perceived barrier* | The possible negative consequences of a certain health action, meaning that the individual will be less likely to adopt preventative behavior if the cost is too high. | The message content addresses and reduce barriers through reassurance, correction of misinformation,  and assistance. | لقاح كورونا للأطفال : أمن...متاح  #جاء ـ دورنا  #لاـيخدعونك ، المعلومات الزائفه لن تنتهي | COVID-19 vaccine for children: Safe... Available.  #It's_our_turn_now  #Don’t_Be_Fooled, misinformation won’t end |
| *Perceived susceptibility* | This refers to beliefs about the likelihood and possibility of developing a disease. | The message content defines the population at risk and their risk levels, personalizes risk based on a person’s trait or behavior, and heightens perceived susceptibility if low. | " الأشخاص الذين لم يتلقوا التطعيم بعد تعافيهم أكثر عرضة للإصابة بفيروس كورونا مرة أخرى أكثر من الذين أكملو جرعات التطعيم " | People who have not been vaccinated after recovery are more likely to get infected with COVID-19 again compared to those who have completed their vaccination doses. |
| *Cues to action/efficacy* | cues that can trigger an action and activate one’s “readiness”/ Refers to an individual feeling confident about taking action through preventative behavior. | The message content  promotes awareness,  provides reminders, how-to information  motivation, training, and guidance in performing an action. | “ جاء دورنا نحن الأطفال الصغار لأخذ اللقاح ، لحجز موعد طفلك توجه لتطبيق صحتي "  " سمو أمير منطقة عسير يتلقى الجرعة الأولي من لقاح كورونا ( كوفيد – ١٩)" | It’s our turn, the young children, to take the vaccine. To book an appointment for your child, go to the ‘Sehhaty’ app.  His Highness the Prince of Asir Region receives the first dose of the COVID-19 vaccine. |
